# Supplementary figures and images for: Metabolic marker gene mining provides insight in global mcrA diversity and, coupled with targeted genome reconstruction, sheds further light on metabolic potential of the Methanomassiliicoccales
Source: PeerJ. 2018 Sep 17;6:e5614. doi: 10.7717/peerj.5614 (PMC6147122; doi:10.7717/peerj.5614)

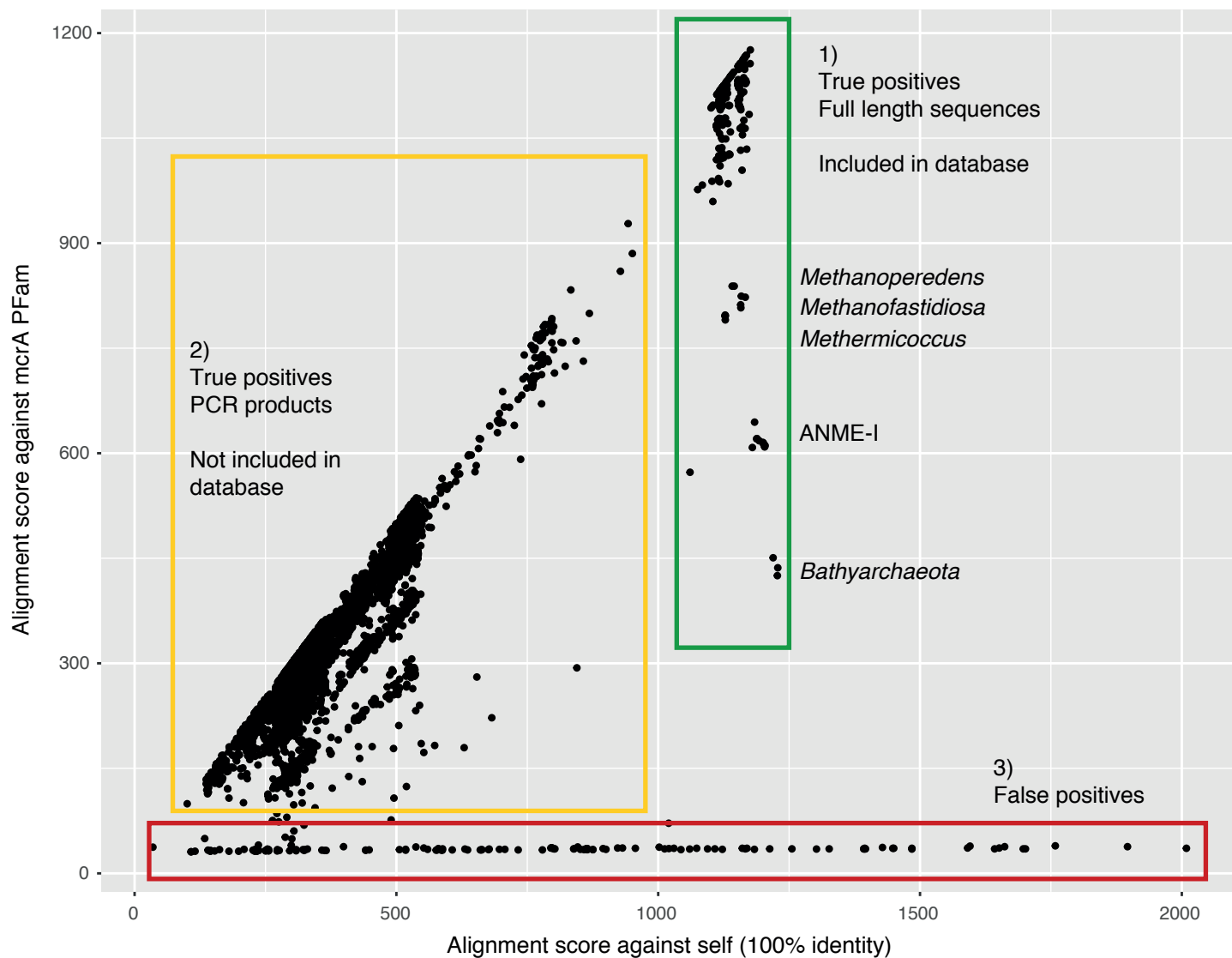

Supplement: Figure S1 — Scatter plot of all sequences in the NCBI-nr database with a hit against the combined PFAM families PF02745 and PF02249. Each dot represents a query sequence, with the maximum possible alignment bitscore (of a self hit) on the x-axis and the alignment bitscore against the PFAM families on the y-axis. Sequences included in the reference database for metagenome mining are boxed in green, sequences used for the amplicon analyses shown in Fig. 2 and Fig. S2 are boxed in yellow and false positives are boxed in red. [file peerj-06-5614-s001.pdf]

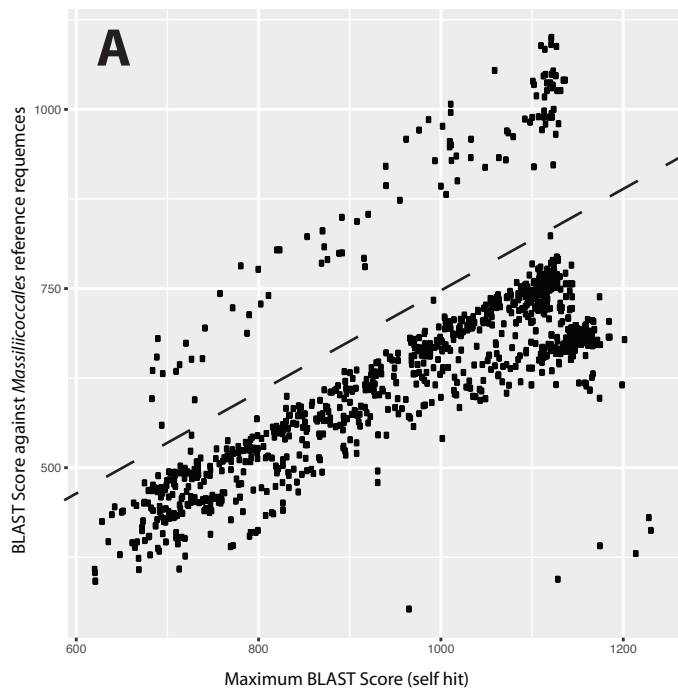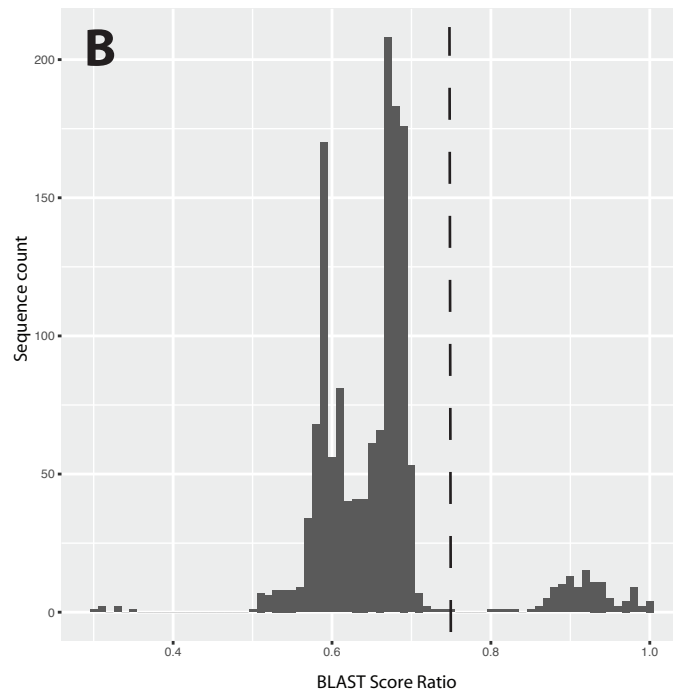

Supplement: Figure S2 — (A) Dot plot comparing the BLAST scores of the reconstructed mcrA amino acid sequences against the 5 reference Methanomassiliicoccales reference sequences (WP_019176774.1; KQM10793.1; WP_058747716.1; WP_048111444.1; AMK13668.1) against the maximum possible score (of a self hit) for each reconstructed sequence. (B) Histogram of the BLAST Score Ratios of the scores shown in (A). Dashed lines represent the cutoff for inclusion the environmental distribution analysis. [file peerj-06-5614-s002.pdf]

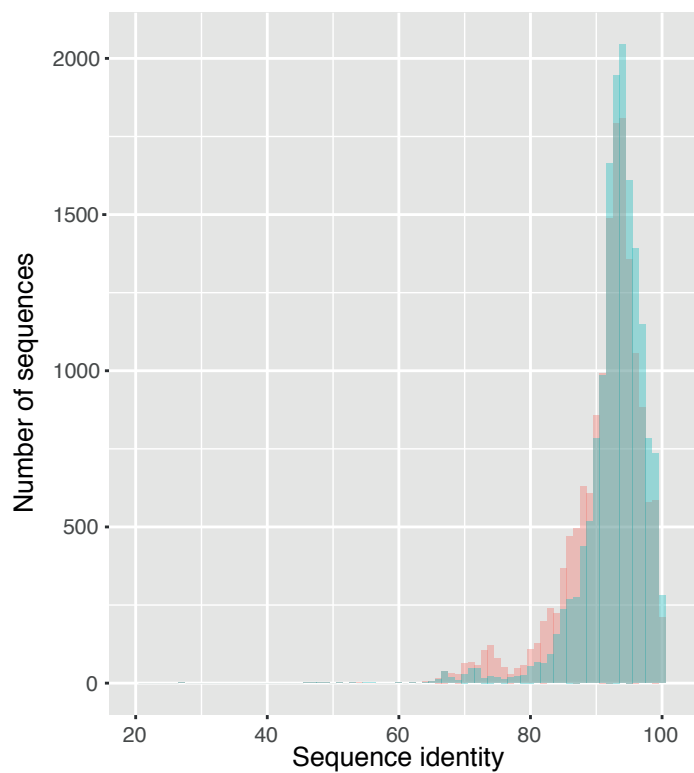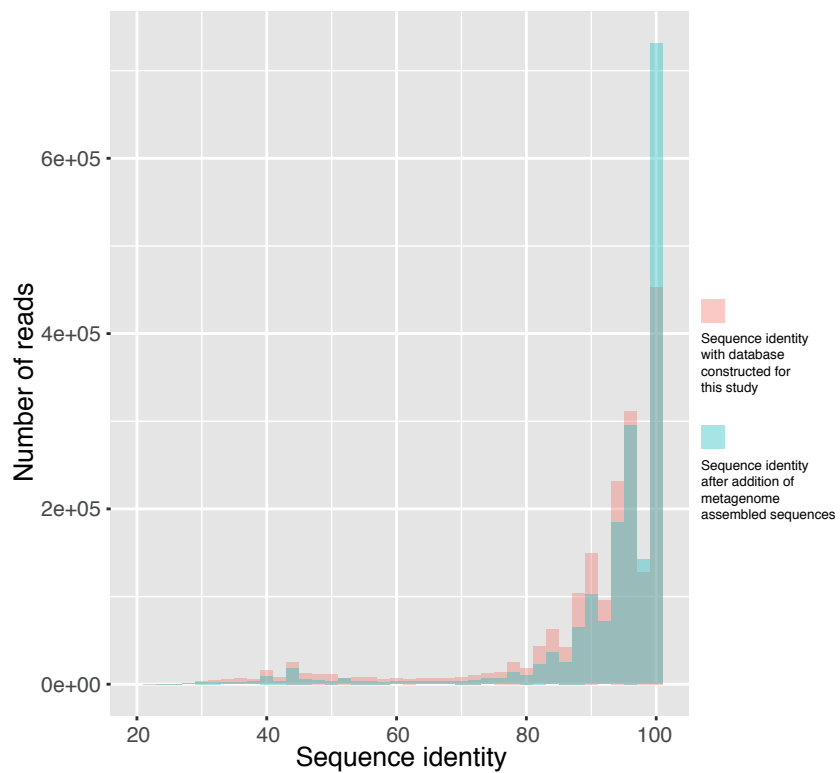

Supplement: Figure S3 — Histograms showing the sequence identity of (A) PCR amplified sequences present in the NCBI-nr, and (B) metagenome reads, after BLAST Score Ratio (BSR) filtering, that match the mcrA database constructed for read recruitment in this study, consisting of 69 mcrA sequences (shown in red), and matching the sequence set after addition of the sequences assembled from metagenomes (shown in green). The rightward shift shows the improved representation of global mcrA diversity, but the remaining low identity sequences indicates more diversity is yet to be discovered. [file peerj-06-5614-s003.pdf]

# A

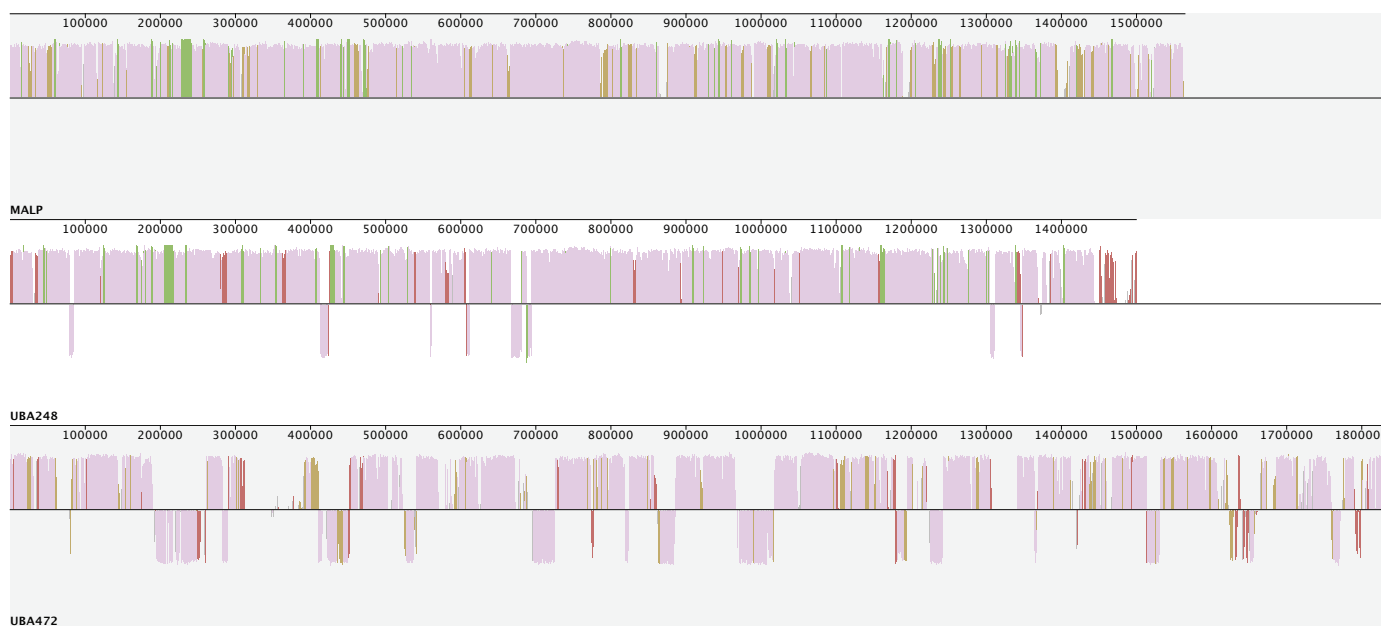

# B

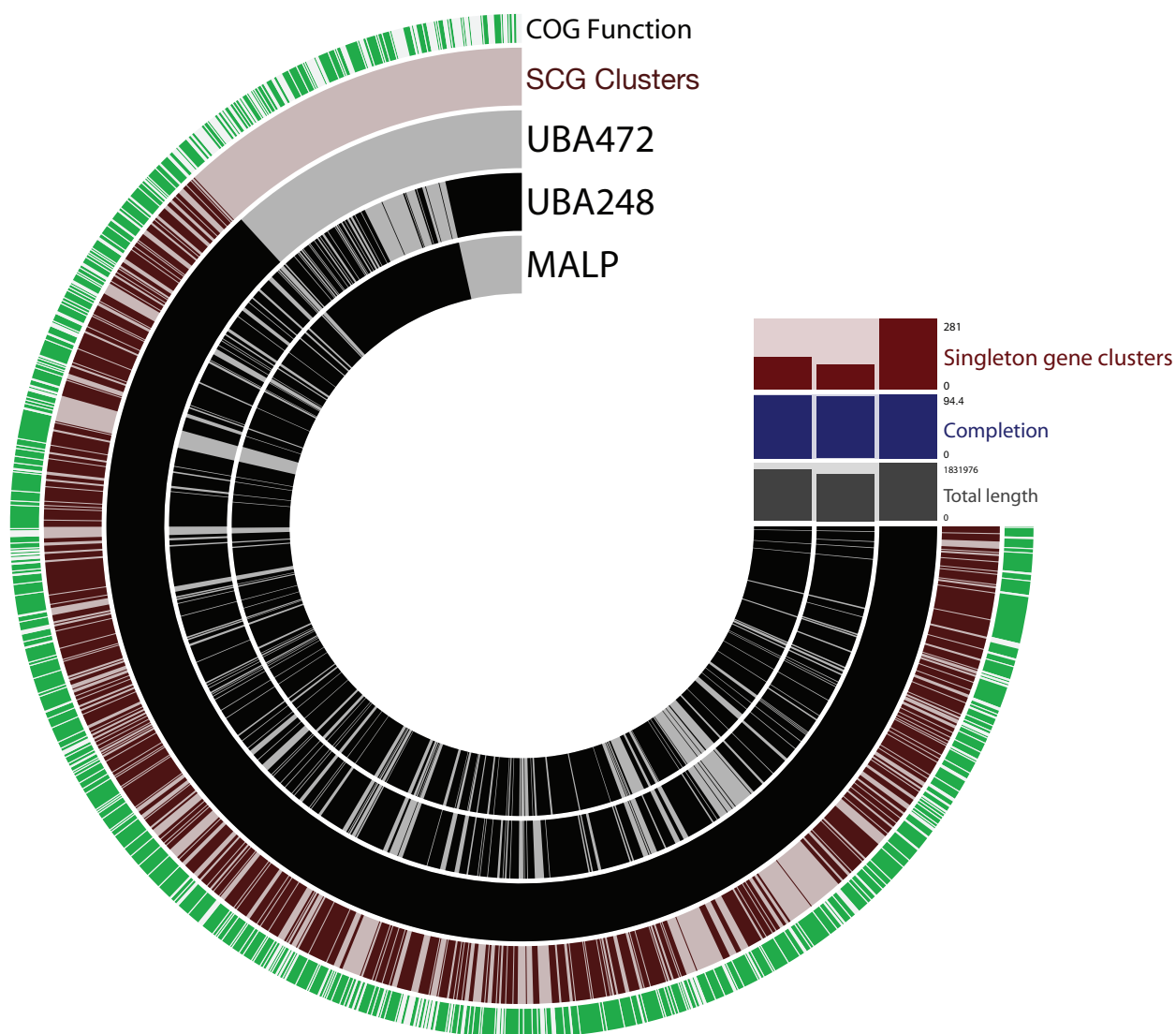

Supplement: Figure S4 — (A) Mauve alignment of the contigs (DNA) comprising the MALP (top) and UBA248 (middle) and UBA472 (bottom) metagenome assembled genomes (MAGs). Purple coloring indicates content present in all three MAGs, green is shared by only MALP and UBA248, yellow is shared by only MALP and UBA472, and red is shared by UBA248 and UBA472. The height of the colored bar reflects average identity of the shared region, between 2 (or 3 in the case of purple) genomes. Numbered scales represent the cumulative number of bases in the contigs of each MAG. (B) Gene content comparison of the three genomes using anvi’o. The inner 3 rings indicate the three genomes, with black indicating the presence of a gene, and gray its absence. The red ring indicates whether a gene is present in all three genomes, in single copy. The green ring indicates the presence (green), or absence (white) of a COG annotation for the gene. [file peerj-06-5614-s004.pdf]
